# Isolation of a *Leuconostoc mesenteroides* Strain With Anti-Porcine Epidemic Diarrhea Virus Activities From Kefir Grains

**DOI:** 10.3389/fmicb.2020.01578

**Published:** 2020-07-15

**Authors:** Wan-Ping Chang-Liao, An Lee, Yu-Han Chiu, Hui-Wen Chang, Je-Ruei Liu

**Affiliations:** ^1^ Institute of Biotechnology, National Taiwan University, Taipei, Taiwan; ^2^ School of Veterinary Medicine, National Taiwan University, Taipei, Taiwan; ^3^ Department of Animal Science and Technology, National Taiwan University, Taipei, Taiwan; ^4^ Center for Biotechnology, National Taiwan University, Taipei, Taiwan; ^5^ Agricultural Biotechnology Research Center, Academia Sinica, Taipei, Taiwan

**Keywords:** *Leuconostoc mesenteroides*, antiviral activity, kefir, porcine epidemic diarrhea virus, interferon-dependent genes

## Abstract

Swine grown under commercial conditions are vulnerable to environmental exposure to several viruses, which may cause infectious diseases and spread easily and rapidly, resulting in significant economic losses in animal husbandry. Previous studies have suggested that probiotics seem to be a new and promising alternative to vaccinations to protect animals against potential viral infections. In this study, we used the Vero cell culture model of infection to study porcine epidemic diarrhea virus (PEDV). We screened lactic acid bacteria (LAB) with anti-PEDV potential from kefir grains, which are starter cultures used to ferment milk into kefir. Twenty-nine LAB strains were isolated and identified as *Enterococcus durans*, *Lactobacillus kefiri*, *Lactococcus lactis*, and *Leuconostoc mesenteroides*, according to 16S ribosomal RNA (rRNA) and *rpo*A gene sequence analyses. The anti-PEDV activities of the LAB intracellular extracts were compared, and the intracellular extracts of *Ln. mesenteroides* showed higher anti-PEDV activities than that of the other species. Among the *Ln. mesenteroides* strains, a strain designated YPK30 showed a higher growth rate than that of the other strains and was further evaluated for its anti-PEDV activity. The results showed that the intracellular extracts of *Ln. mesenteroides* YPK30 possessed *in vitro* prophylactic, therapeutic, and direct-inhibitory effects against PEDV in the Vero cell model. The expression levels of Type 1 interferon (IFN)-dependent genes, including Myxovirus resistance 1 (*MX1*) and interferon-stimulated gene 15 (*ISG15*), were significantly increased after treatment with intracellular extracts of *Ln. mesenteroides* YPK30 for 24 h. Such expression suggests that the anti-PEDV activity of *Ln. mesenteroides* YPK30 could be attributed to its up-regulatory effect on the expression of *MX1* and *ISG15* genes. These results suggested that *Ln. mesenteroides* YPK30 has the potential to provide some levels of host protection against PEDV infections.

## Introduction

In order to increase swine and poultry production, it is common to raise animals in high-density populations. Swine grown under commercial conditions are vulnerable to environmental exposure to several viruses. Some viruses may cause infectious diseases, which are spread easily and rapidly cause significant economic losses in animal husbandry. Vaccination is one of the most efficient strategies to prevent viral diseases and control infections and is the current industry standard. Efficacious vaccines have been developed and applied successfully for the prevention of several infectious viral diseases in swine, such as porcine parvovirus (PPV), foot-and-mouth disease virus (FMDV), porcine circovirus type 2 (PCV2), and transmissible gastroenteritis virus (TGEV; Meng, 2012; Gerdts and Zakhartchouk, 2017). However, some of the vaccination methods require the operator to handle each animal, induce the stress on the animal, and are time-consuming and costly (Marangon and Busani, 2007). Although efficacious vaccines are available to reduce the impact of the infectious viruses mentioned above, unfortunately, substantial challenges remain in obtaining safe and efficacious vaccines for a variety of newly emerging and re-emerging viruses, such as African swine fever virus and porcine epidemic diarrhea virus (PEDV; Lee, 2015). PEDV is a member of the genus *Alphacoronavirus* in the family *Coronaviridae* of the order Nidovirales and has emerged as a significant pathogen causing lethal watery diarrhea, vomiting, and dehydration in nursing piglets. Highly pathogenic strains of PEDV have mortality rates of 50–90% in neonatal piglets, which has resulted in huge economic losses to the swine industry worldwide (Koonpaew et al., 2019; Wang et al., 2019). Accumulated evidence indicates that PEDV encodes defensive mechanisms to evade virus recognition by host pattern recognition receptors (PRRs) present on antigen-presenting cells, inhibit interferon (IFN) induction, and antagonize IFN signaling and antiviral effector machinery (Hao et al., 2019; Koonpaew et al., 2019). PEDV has been studied extensively and some vaccines have been developed against PEDV, but the efficacy of these vaccines in the field remains questionable (Wang et al., 2019).

Probiotics, which are live microorganisms that when administered in adequate amounts confer a health benefit on the host, have long been used as feed additives because of their abilities to normalize gut microbiota, boost the immune system, prevent diarrhea, and improve feed conversion efficiency (Fontana et al., 2012; Alonso and Guarner, 2013). The effect of probiotics is achieved mainly through the intervention on gut microbiota, which increases the levels of beneficial bacteria and decreases the pathogenic populations in the gastrointestinal tract (Liu et al., 2015; Yadav and Shukla, 2019). In addition to the microbiota-modulatory properties, recent studies showed that the immunomodulatory activity of probiotics is another important mechanism of action of probiotics for the inhibition of pathogens (Llewellyn and Foey, 2017; Azad et al., 2018). Those immunoregulatory probiotics provide host protection against pathogenic infections by modulating innate and adaptive antiviral immune responses (Villena et al., 2016). Several probiotic strains, most of them belonging to *Lactobacillus* and *Bifidobacterium* genera, have been shown to perform antiviral activities (Villena et al., 2016; Arena et al., 2018). These antiviral activities could be mediated by the immunomodulatory properties of probiotics because it was observed that administration of probiotics induced the expression of IFN and interferon-stimulated genes (ISGs), which are crucial components of the IFN responses and play a key role in establishing an antiviral state for virus clearance and restriction of spread (Zelaya et al., 2015; Villena et al., 2016; Arena et al., 2018; Eguchi et al., 2019). If probiotics have antiviral activity, it seems to be a new and promising alternative to vaccinations to protect animals against potential viral infections (Al Kassaa et al., 2014).

Kefir is an acidic and mildly alcoholic fermented milk product that is believed to have many beneficial activities, such as hypocholesterolemic activity, antibacterial and antifungal activities, antitumor activity, immunomodulatory activity, and quickening of wound healing (Bourrie et al., 2016). Traditionally, kefir is produced from milk fermented with a mixed microflora confined to a matrix of discrete kefir grains, which are a combination of bacteria and yeasts in a symbiotic matrix (Marshall and Cole, 1985). Various bacteria and yeasts have been identified in kefir grains. The bacteria present in kefir grains may include *Acetobacter*, *Bifidobacterium*, *Lactobacillus*, *Lactococcus*, *Leuconostoc*, *Oenococcus*, and *Streptococcus*, while the yeasts present in kefir grains may include *Candida*, *Kluyveromyces*, and *Pichia* (Lin et al., 1999; Wang et al., 2012; Bourrie et al., 2016). Numerous bacterial strains with specific properties, such as hypocholesterolemic effect, antiallergenic effect, immunoregulatory effects, and antipathogenic activities, have been isolated from kefir grains (Prado et al., 2015). However, to the best of our knowledge, following a thorough review of the relevant literature, there has been no study to isolate bacterial strains with antiviral activities from kefir grains.

In the present study, we used the Vero cell culture model of infection by PEDV to screen lactic acid bacteria (LAB) with anti-PEDV activities from kefir grains. The anti-PEDV activities of the LAB strains were evaluated in prophylactic, therapeutic, and direct-inhibitory models. The strains with anti-PEDV activities were further studied to define their effects on the expression of Type 1 IFN-dependent genes, including 2'-5'-oligoadenylate synthetase 1 (*OAS1*), Myxovirus resistance 1 (*MX1*), and interferon-stimulated gene 15 (*ISG15*) in Vero cells.

## Materials and Methods

### Isolation of LAB Strains From Kefir Grains

A total of 29 LAB strains were isolated from kefir grains according to the method described by Lin et al. (1999). The LAB isolates were cultured in de Mann, Rogosa, Sharpe (MRS) broth (Oxoid, Basingstoke, UK) at 37°C for 16 h without shaking. The bacterial concentrations were measured by optical density readings at 600 nm or by traditional colony counting methods (Vinderola et al., 2000).

### Identification of the LAB Isolates

For colony morphological observation, the LAB isolates were cultivated on MRS agar plates (Oxoid) or blood agar plates (Merck, Darmstadt, Germany) at 37°C for 24 h and then observed. For cell morphological observation, the LAB isolates were cultured in MRS broth (Oxoid) at 37°C for 16 h without shaking. The bacterial cells were harvested by centrifugation at 5,000*g* for 20 min at 4°C, stained with Gram stain (Sigma-Aldrich, St. Louis, MO, USA) according to the manufacturer’s instructions, and observed under a microscope. Unstained bacteria were observed under a phase-contrast microscope.

Molecular identification of the bacterial isolates was performed by the methods described by Weisburg et al. (1990) and Naser et al. (2005). Genomic DNA of the bacterial isolates was isolated using the DNeasy Blood & Tissue kit (Qiagen Inc., Valencia, CA, USA). The standard 16S ribosomal RNA (rRNA) gene primers and RNA polymerase α subunit gene *rpo*A primers were used for PCR to amplify the 16S rRNA gene and *rpo*A gene sequences of the bacterial isolates, respectively (Weisburg et al., 1990; Naser et al., 2005). The resultant PCR products were sequenced by an automatic sequencing service provided by Genomics Biotech Inc., (New Taipei City, Taiwan). The nucleotide sequences of the 16S rRNA and *rpo*A genes were aligned using the National Center for Biotechnology Information’s Basic Local Alignment Search Tool (BLAST).

For the determination of biochemical characteristics, the carbon source use profiles of the LAB isolates were determined using an API 50 CH system (bioMerieux, Inc., Marcy l’Etoile, France) according to the manufacturer’s instructions. For the determination of physiological characteristics, the LAB isolates were cultured at 10°C, 37°C, pH 4.8, or in the presence of 10% ethanol according to the methods described by Lin et al. (1999).

### Cytotoxicity Assay of the LAB Isolates

Before the anti-PEDV activity assay, the cytotoxicity of the LAB isolates on the African green monkey kidney cell line Vero was evaluated according to the method described by Mosmann (1983). The Vero C1008 cells were purchased from the Bioresource Collection and Research Center of Food Industry Research and Development Institute (BCRC, Hsinchu, Taiwan) and were routinely grown at 37°C in a humidified atmosphere of 95% air and 5% CO_2_ in Dulbecco’s modified Eagle’s medium (DMEM, Gibco, Grand Island, NY, USA) supplemented with 10% fetal bovine serum (FBS; Moregate Biotech., Queensland, Australia).

For evaluation of cytotoxicity, a 1.0-ml aliquot of the 16-h culture of each bacterial strain was centrifuged at 9,000*g* for 10 min at 4°C. The bacteria were collected, washed twice with sterile phosphate-buffered saline (PBS; 0.1 M, pH 7.0), resuspended in 1.0 ml of sterile PBS, and sonicated for 10 min with an ultrasonicator (Model XL, Misonix, Farmingdale, NY). The sonicated bacteria were fractioned into intracellular extracts and cell-wall pellet fractions by subsequent centrifugation at 13,000*g* for 20 min at 4°C. The intracellular extracts were harvested, and the cytotoxicity on Vero cells was determined using the 3-(4,5-dimethylthiazol-2-yl)-2,5-diphenyltetrazolium bromide (MTT) assay according to the method described by Mosmann (1983). Briefly, Vero cells were seeded at a density of 1.5 × 10^5^ cells/well on a 24-well plate in 500 μl of DMEM. After incubating at 37°C for 24 h, 100 μl of the bacterial intracellular extracts were added into each well and incubated at 37°C for another 24 h. After washing twice with sterile PBS, the cells were incubated with 500 μl of MTT (5 mg/ml in PBS) at 37°C for 2 h. After the incubation, the medium was removed, and 200 μl of dimethyl sulfoxide (DMSO) were added into each well to dissolve the formazan crystals. The absorbance was measured at 570 nm using a microplate reader (Victor^3^, PerkinElmer Inc., Waltham, MA, USA), and percentages of cell metabolic activity were calculated as follows:

$$\begin{array}{l} \mathrm{Metabolic activity}\;\left(\%\right)=\mathrm{Absorbance of Sample}/\\ \;\;\mathrm{Absorbance of Control}\times 100 \end{array}$$

where the Absorbance of Sample is the absorbance of cells treated with test sample and the Absorbance of Control is the absorbance of cells treated with DMSO.

### Assessment of the *in vitro* Prophylactic Effects of LAB Against PEDV

The PEDV Taiwan Pintung 52 strain was isolated in early 2014 from the intestinal homogenate of a 7-day-old suckling pig in Taiwan and adapted to Vero cells as previously described by Chang et al. (2017). Viral infection and propagation were performed in Vero cells according to the method described by Chang et al. (2017).

Before the anti-PEDV activity screening experiments were conducted, the viral titers of PEDV were adjusted to 200 fifty-percent tissue culture infective dose (TCID_50_)/ml, and the intracellular extracts of bacterial isolates were prepared as described above. Vero cells were seeded at a density of 3 × 10^4^ cells/well on a 96-well plate in 100 μl of modified postinoculation (PI) medium containing DMEM (Gibco, Grand Island, NY, USA) supplemented with tryptose phosphate broth (0.3%), yeast extract (0.02%), and 10 μg/ml of trypsin. After incubating at 37°C for 24 h, 100 μl of the bacterial intracellular extracts were added into each well and incubated at 37°C for another 24 h. After washing the cells twice with PI medium, 200 μl of PI medium containing 200 TCID_50_/ml of the PEDV was added into each well and incubated at 37°C for 1 h. After 1 h of incubation, the supernatants were replaced by fresh PI medium and the cells were maintained at 37°C for 48 h. After washing twice with sterile PBS (0.1 M, pH 7.0), the cell metabolic activity was determined using the MTT assay as described previously.

### Assessment of the *in vitro* Prophylactic Effects of LAB Intracellular Extract, Cell-Wall Fraction, and Extracellular Supernatant Against PEDV

A 1.0-ml aliquot of the 16-h culture of each LAB strain was centrifuged at 9,000*g* for 10 min at 4°C. The resultant extracellular supernatants and bacterial cells were harvested separately. The bacterial cells were washed twice with sterile PBS, resuspended in 1.0 ml of sterile PBS, and sonicated for 10 min with an ultrasonicator (Model XL, Misonix, Farmingdale, NY, USA). The sonicated bacterial cells were fractioned into intracellular extracts and cell-wall pellet fractions by subsequent centrifugation at 13,000*g* for 20 min at 4°C. The extracellular supernatants, intracellular extracts, and cell-wall fractions of each bacterial strain were harvested, and the anti-PEDV activities were evaluated as described above.

### Assessment of the *in vitro* Therapeutic Effects of LAB Against PEDV

Vero cells were seeded on a 96-well plate and incubated at 37°C for 24 h. Afterward, the cells were washed with PI medium, and 200 μl of PI medium containing 200 TCID_50_/ml of PEDV were added into each well and incubated at 37°C for 1 h. After 1 h of incubation, the supernatants were replaced by 100 μl of fresh PI medium and 100 μl of the bacterial intracellular extracts. The cells were maintained at 37°C for 48 h. After washing twice with sterile PBS, the cell metabolic activity was determined by MTT assay as described previously.

### Assessment of the *in vitro* Direct-Inhibitory Effects of LAB Against PEDV

Vero cells were seeded on a 96-well plate and incubated at 37°C for 24 h. After the cells were washed twice with PI medium, 100 μl of the bacterial intracellular extracts and 100 μl of PI medium containing 200 TCID_50_/ml of PEDV were added into each well and incubated at 37°C for 1 h. After 1 h of incubation, the supernatants were replaced by 200 μl of fresh PI medium and the cells were maintained at 37°C for 48 h. After washing twice with sterile PBS, the cell metabolic activity was determined by MTT assay as described previously.

### Quantitative Reverse-Transcription PCR for Quantification of Type 1 IFN-Dependent Genes

The expression levels of Type 1 IFN-dependent genes, including *OAS1*, *MX1*, and *ISG15*, were quantified in Vero cells. Vero cells were seeded and incubated at 37°C for 24 h. After washing twice with sterile PBS, the Vero cells were treated with the bacterial intracellular extracts, human IFN-α2b, or PBS and incubated at 37°C for up to 48 h. During the incubation, the Vero cells were harvested at 0, 24, or 48 h, and total RNA was extracted by using TRizol reagent (Invitrogen, Carlsbad, CA, USA). Residual DNA was removed with RNase-free DNase I (Invitrogen) treatment. RNA concentrations were measured in triplicate using a NanoDrop spectrophotometer (NanoDrop Technologies Inc., Wilmington, DE, USA). Complementary DNA (cDNA) was prepared from total RNA with a High-Capacity cDNA Reverse Transcription Kit (Applied Biosystems, Foster City, CA, USA) with random hexamer primers. The gene expression analysis was done by Quantitative Reverse-Transcription PCR (qRT-PCR) using a KAPA SYBR FAST qPCR Master Mix Kit (Kapa Biosystems, Wilmington, MA, USA) on a LightCycler 480 System (Roche Applied Science, Indianapolis, IN, USA). Glyceraldehyde 3-phosphate dehydrogenase (GAPDH) was chosen as an internal control, and all relative gene expression levels were normalized to GAPDH by the comparative C_T_ method. The primers used for the relative quantification are provided in Table 1.

**Table 1:** Primer list.

Gene	Primer sequence (5'–3')	Application	References
16S ribosomal RNA (rRNA)	Forward: AGAGTTTGATCMTGGCTCAG Reverse: GGTTACCTTGTTACGACTT	PCR	Weisburg et al. (1990)
*rpoA*	Forward: ATGATYGARTTTGAAAAACC Reverse: ACHGTRTTRATDCCDGCRCG	PCR	Naser et al. (2005)
Oligoadenylate synthetase 1 (*OAS1*)	Forward: GGTTGTCTTCCTCAGTCCTC Reverse: AGCCTGGACCTCAAACTTCA	Quantitative reverse-transcription PCR (qRT-PCR)	Shen et al. (2016)
Myxovirus resistance 1 (*MX1*)	Forward: GCAGCCAGTACGAGGAGAAG Reverse: CTCCTGACAGTGCCTCCAAC	qRT-PCR	Shen et al. (2016)
Interferon-stimulated gene 15 (*ISG15*)	Forward: GGGCAACGAGTTCCAGGT Reverse: CACCACCAGCAGGACCGT	qRT-PCR	Shen et al. (2016)
Glyceraldehyde 3-phosphate dehydrogenase (*GAPDH*)	Forward: AGCCAAAAGGGTCATCATCT Reverse: ATGAGTCCTTCCACGATACC	qRT-PCR	Shen et al. (2016)

### Statistical Analysis

The data were analyzed using SPSS version 25 software (IBM SPSS, New York, NY, USA). One-way analysis of variance (ANOVA) followed by Duncan’s multiple range test was used to detect the differences among the means of the different treatment groups, and a value of *p* less than 0.05 was considered significant. Student’s *t*-test was used to detect the differences between the treatment and control groups, and a value of *p* less than 0.05 was considered significant. Each experiment was conducted in triplicate, and all results were expressed as means ± standard deviations.

## Results

### Molecular Identification of the LAB Strains Isolated From Kefir Grains

Twenty-nine LAB strains were isolated from kefir grains. According to the 16S rRNA and *rpo*A gene sequence analysis, three isolated strains belong to the species *Enterococcus durans*, 16 isolated strains might belong to the species *Lactobacillus kefiri*, five isolated strains might belong to the species *Lactococcus lactis*, and five isolated strains might belong to the species *Leuconostoc mesenteroides* (Table 2).

**Table 2:** Identification of bacterial strains based on 16S rRNA and *rpoA* sequence analysis.

Strain ID	Closely related validly published taxa	Sequence similarity of 16S rRNA gene (%)	Sequence similarity of *rpoA* gene (%)
YPK1	*Enterococcus durans*	99.93	100.00
YPK2	*Leuconostoc mesenteroides*	99.86	99.72
YPK3	*Lactobacillus kefiri*	99.79	100.00
YPK4	*Lactobacillus kefiri*	99.86	100.00
YPK5	*Leuconostoc mesenteroides*	100.00	99.58
YPK6	*Lactobacillus kefiri*	100.00	100.00
YPK7	*Lactobacillus kefiri*	99.86	100.00
YPK8	*Lactobacillus kefiri*	99.79	100.00
YPK9	*Lactobacillus kefiri*	99.65	100.00
YPK10	*Lactobacillus kefiri*	99.72	100.00
YPK11	*Lactococcus lactis*	99.64	99.29
YPK12	*Lactobacillus kefiri*	99.79	100.00
YPK13	*Enterococcus durans*	99.93	99.86
YPK14	*Lactobacillus kefiri*	99.72	100.00
YPK15	*Lactobacillus kefiri*	99.72	100.00
YPK16	*Lactococcus lactis*	99.93	100.00
YPK17	*Lactobacillus kefiri*	99.58	100.00
YPK18	*Leuconostoc mesenteroides*	99.79	99.86
YPK19	*Lactococcus lactis*	99.93	99.72
YPK20	*Lactococcus lactis*	99.93	99.57
YPK22	*Lactobacillus kefiri*	99.79	100.00
YPK23	*Lactococcus lactis*	99.93	99.58
YPK24	*Enterococcus durans*	99.93	100.00
YPK25	*Leuconostoc mesenteroides*	100.00	99.86
YPK26	*Lactobacillus kefiri*	99.72	100.00
YPK27	*Lactobacillus kefiri*	100.00	100.00
YPK28	*Lactobacillus kefiri*	99.58	100.00
YPK29	*Lactobacillus kefiri*	99.72	100.00
YPK30	*Leuconostoc mesenteroides*	99.93	99.57

### *In vitro* Prophylactic Effects of LAB Against PEDV

**Figure 1:**
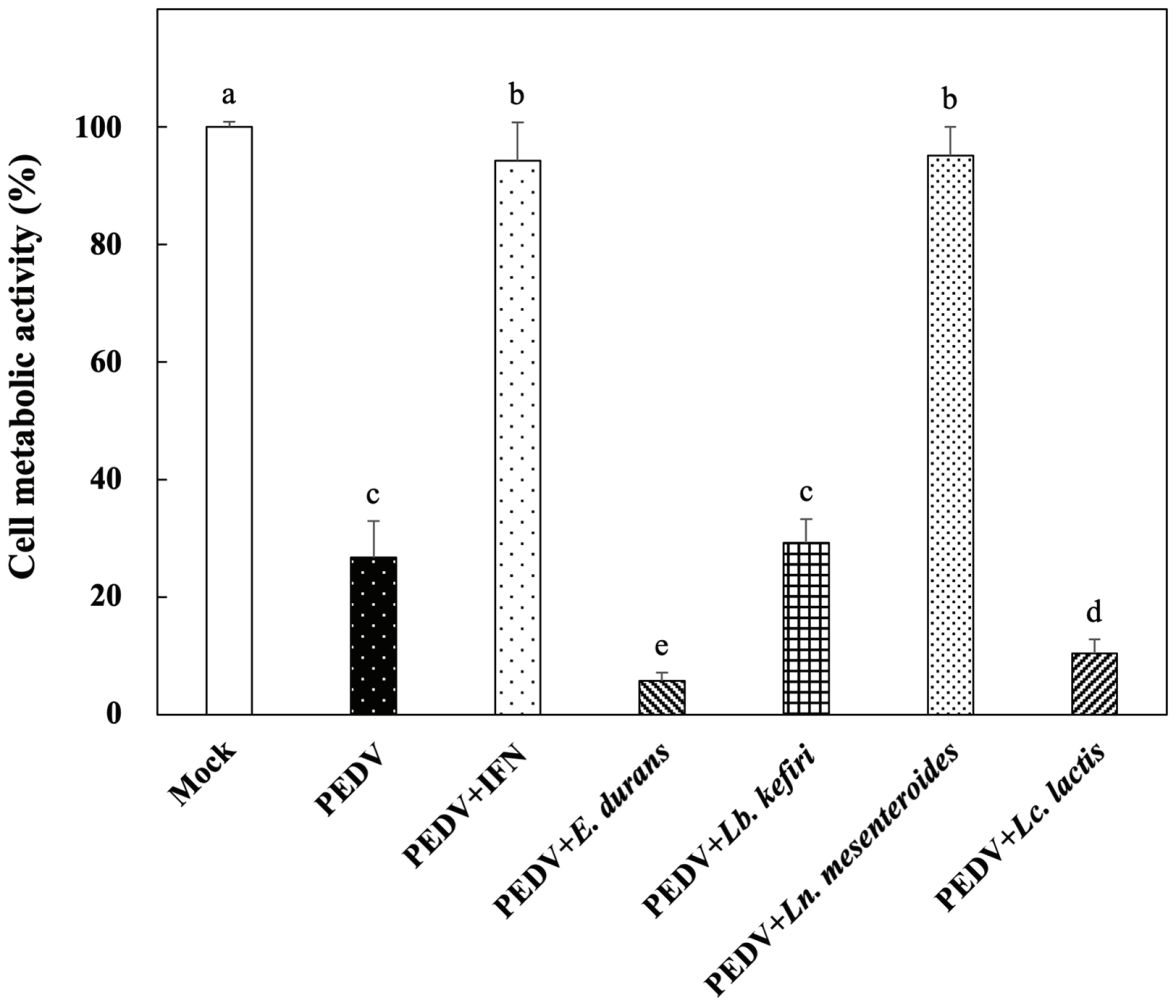
*In vitro* prophylactic effects of the intracellular extracts of lactic acid bacteria isolated from kefir grains against porcine epidemic diarrhea virus (PEDV) infection in Vero cells. Bar chart showing percentage of cell metabolic activity of seven sample conditions: (1) Mock: cells pretreated with phosphate-buffered saline (PBS) and then mock-infected with PBS, (2) PEDV: cells pretreated with PBS and then infected with PEDV, (3) PEDV+IFN: cells pretreated with interferon (IFN)-α2b and then infected with PEDV, (4) PEDV+*E. durans*: cells pretreated with the intracellular extracts of *Enterococcus durans* and then infected with PEDV, (5) PEDV+*Lb. kefiri*: cells pretreated with the intracellular extracts of *Lactobacillus kefiri* and then infected with PEDV, (6) PEDV+*Ln. mesenteroides*: cells pretreated with the intracellular extracts of *Leuconostoc mesenteroides* and then infected with PEDV, and (7) PEDV+*Lc. lactis*: cells pretreated with the intracellular extracts of *Lactococcus lactis* and then infected with PEDV. All data are expressed as mean ± SD (*n* = 3). Bars marked with the same letter are not significantly different (*p* > 0.05).

**Figure 2:**
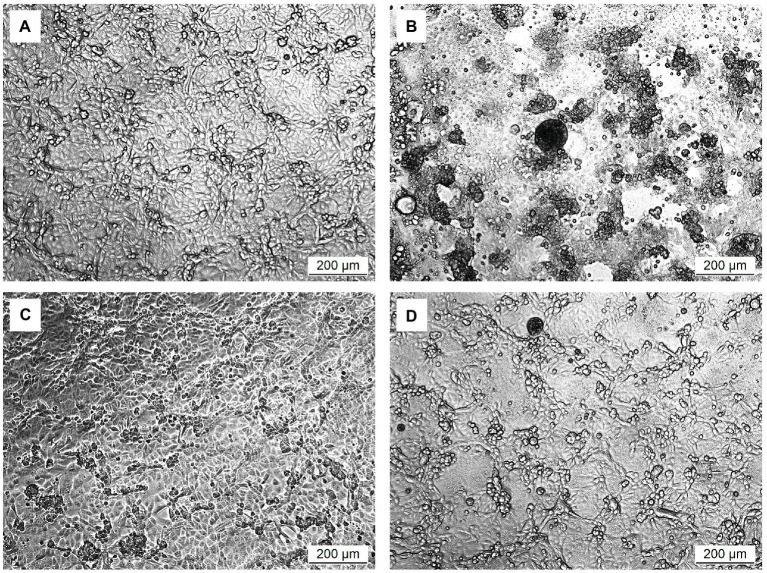
Microscopic observation of the effect of pretreatments on the development of cytopathic effects in Vero cells infected with PEDV. **(A)** Cells pretreated with PBS and then mock-infected with PBS. **(B)** Cells pretreated with PBS and then infected with PEDV. **(C)** Cells pretreated with IFN-α2b and then infected with PEDV. **(D)** Cells pretreated with the intracellular extracts of *Ln. mesenteroides* isolated from kefir grains and then infected with PEDV.

The *in vitro* prophylactic effects of the LAB strains on PEDV were evaluated in the Vero cell model. Vero cells were pretreated with the intracellular extracts of LAB for 24 h. After the removal of the bacterial intracellular extracts, the Vero cells were infected with PEDV. If the intracellular extracts of LAB possessed an *in vitro* prophylactic effect against PEDV, the bacterial pretreated Vero cells would be infected with less PEDV and thus would show higher cell metabolic activity than the un-pretreated cells. The *in vitro* prophylactic effects of the bacterial intracellular extracts of different LAB species against PEDV were compared, and human IFN-α2b was used as a positive control. Vero cells pretreated with IFN-α2b prior to PEDV infection showed a significantly higher cell metabolic activity and less cytopathic effects than the un-pretreated cells (Figures 1, 2), indicating that IFN-α2b possessed an *in vitro* prophylactic effect against PEDV. Among the cells pretreated with the intracellular extracts of different LAB species, the cells pretreated with the intracellular extracts of *Ln. mesenteroides* showed significantly higher cell metabolic activity than those pretreated with the intracellular extracts of the other LAB species (Figure 1). In addition, Vero cells pretreated with the intracellular extracts of *Ln. mesenteroides* also showed less cytopathic effects than the un-pretreated cells (Figure 2), indicating that *Ln. mesenteroides* possessed an *in vitro* prophylactic effect against PEDV.

### *In vitro* Prophylactic Effects of *Ln. mesenteroides* Strains Against PEDV

**Figure 3:**
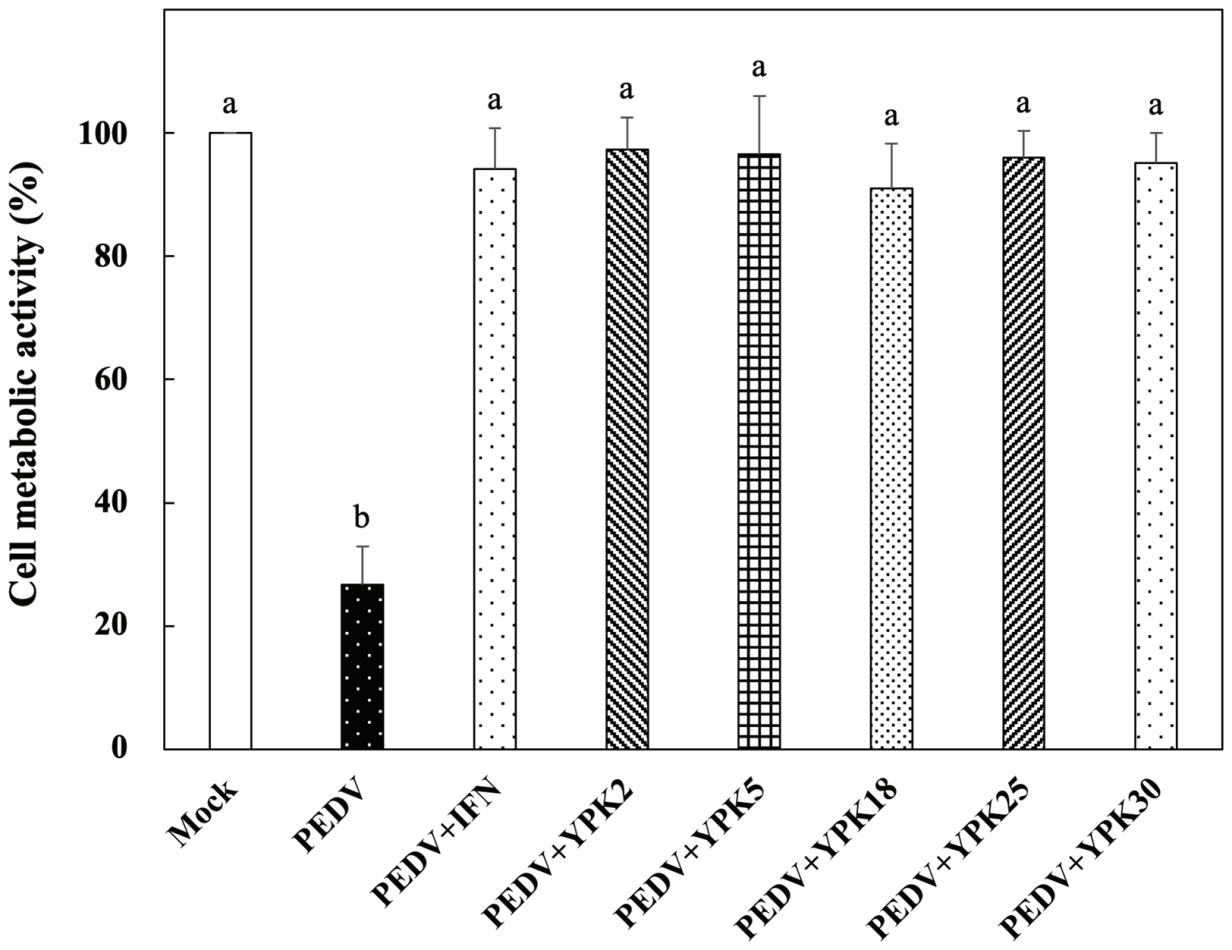
*In vitro* prophylactic effects of intracellular extracts of different *Ln. mesenteroides* strains isolated from kefir grains against PEDV infection in Vero cells. Bar chart showing percentage of cell metabolic activity of eight sample conditions: (1) Mock: cells pretreated with PBS and then mock-infected with PBS, (2) PEDV: cells pretreated with PBS and then infected with PEDV, (3) PEDV+IFN: cells pretreated with IFN-α2b and then infected with PEDV, (4) PEDV+YPK2, (5) PEDV+YPK5, (6) PEDV+YPK18, (7) PEDV+YPK25, and (8) PEDV+YPK30: cells pretreated with the intracellular extracts of *Ln. mesenteroides* YPK2, YPK5, YPK18, YPK25, and YPK30 strains, respectively, and then infected with PEDV. All data are expressed as mean ± SD (*n* = 3). Bars marked with the same letter are not significantly different (*p* > 0.05).

The *in vitro* prophylactic effects of the five strains of *Ln. mesenteroides* isolated from kefir grains on PEDV were further compared with each other. As shown in Figure 3, the metabolic activity of Vero cells pretreated with the intracellular extracts of *Ln. mesenteroides*, regardless of which strain, were similar to those pretreated with IFN-α2b (*p* > 0.05) but were significantly higher than the un-pretreated cells (*p* < 0.05), indicating that all the *Ln. mesenteroides* strains isolated from kefir grains possessed *in vitro* prophylactic effects against PEDV.

### *In vitro* Prophylactic Effects of *Ln. mesenteroides* YPK30 Intracellular Extract, Cell-Wall Fraction, and Extracellular Supernatant Against PEDV

**Figure 4:**
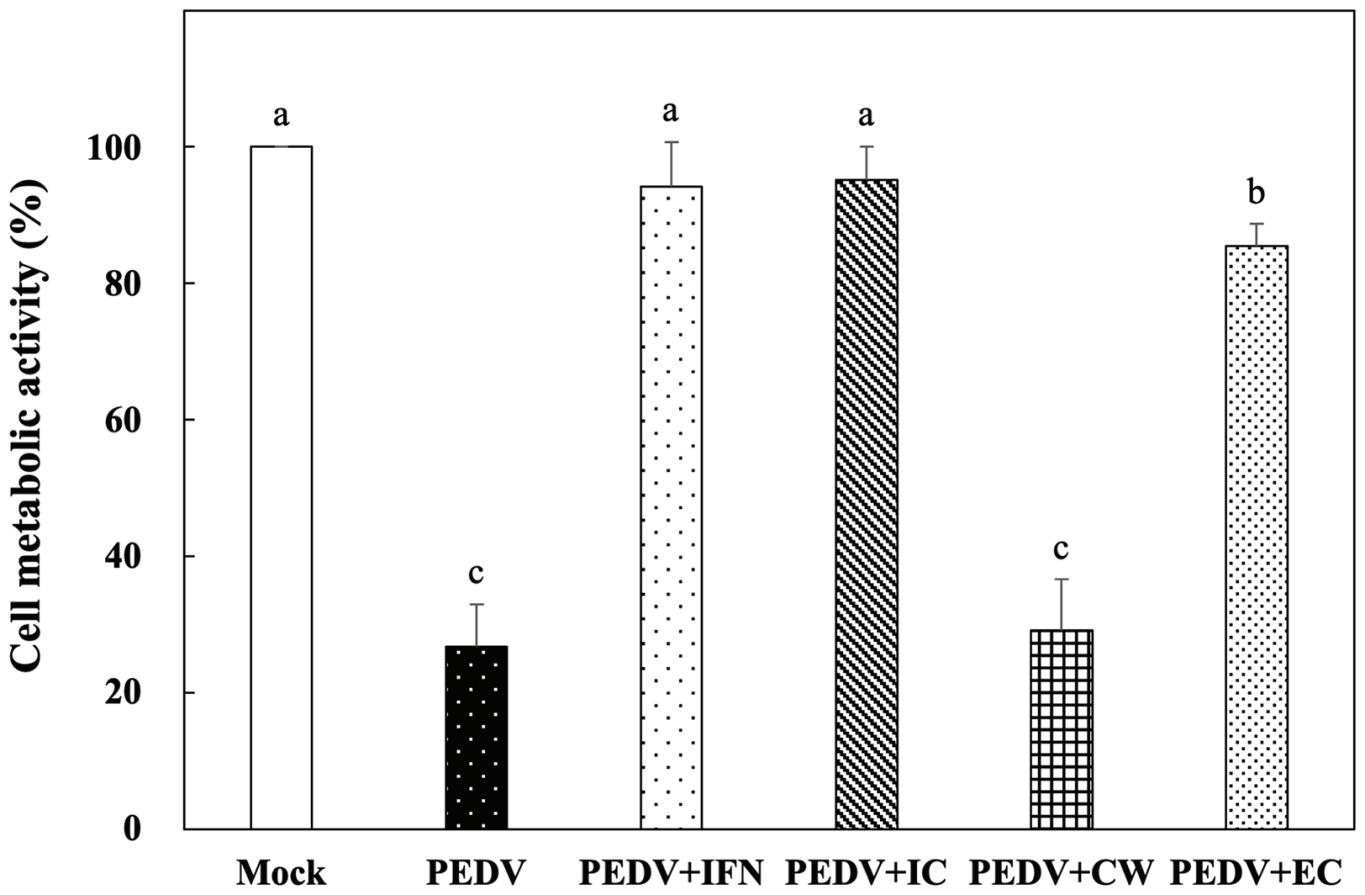
*In vitro* prophylactic effects of intracellular extracts, cell-wall fractions, or extracellular supernatants of *Ln. mesenteroides* YPK30 against PEDV infection in Vero cells. Bar chart showing percentage of cell metabolic activity of six sample conditions: (1) Mock: cells pretreated with PBS and then mock-infected with PBS, (2) PEDV: cells pretreated with PBS and then infected with PEDV, (3) PEDV+IFN: cells pretreated with IFN-α2b and then infected with PEDV, (4) PEDV+IC: cells pretreated with the intracellular extracts of *Ln. mesenteroides* YPK30 and then infected with PEDV, (5) PEDV+CW: cells pretreated with the cell-wall fractions of *Ln. mesenteroides* YPK30 and then infected with PEDV, and (6) PEDV+EC: cells pretreated with the extracellular supernatants of *Ln. mesenteroides* YPK30 and then infected with PEDV. All data are expressed as mean ± SD (*n* = 3). Bars marked with the same letter are not significantly different (*p* > 0.05).

Among the *Ln. mesenteroides* strains, a strain designated YPK30 showed a higher growth rate than the other strains (data not shown) and was further evaluated for its basis of anti-PEDV activity. We compared the *in vitro* prophylactic effects of intracellular extracts, cell-wall fractions, and extracellular supernatants of YPK30 against PEDV. As shown in Figure 4, the metabolic activity of Vero cells pretreated with the intracellular extracts and extracellular supernatants of YPK30 were similar to each other and were significantly higher than those of un-pretreated cells or those pretreated with the cell-wall fractions of YPK30 (*p* < 0.05), indicating that both of the intracellular extracts and extracellular supernatants of YPK30 possessed *in vitro* prophylactic effects against PEDV.

### *In vitro* Therapeutic Effects of *Ln. mesenteroides* YPK30 Intracellular Extract Against PEDV

**Figure 5:**
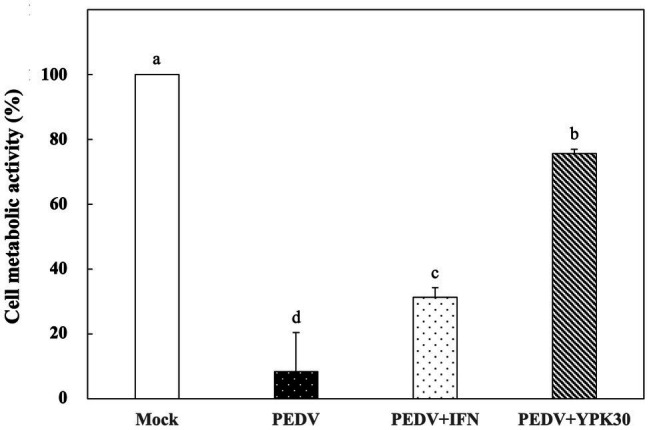
*In vitro* therapeutic effects of the intracellular extracts of *Ln. mesenteroides* YPK30 against PEDV infection in Vero cells. Bar chart showing percentage of cell metabolic activity of four sample conditions: (1) Mock: cells mock-infected with PBS and then treated with PBS, (2) PEDV: cells infected with PEDV and then treated with PBS, (3) PEDV+IFN: cells infected with PEDV and then treated with IFN-α2b, and (4) PEDV+YPK30: cells infected with PEDV and then treated with the intracellular extracts of *Ln. mesenteroides* YPK30. All data are expressed as mean ± SD (*n* = 3). Bars marked with the same letter are not significantly different (*p* > 0.05).

Vero cells were infected with PEDV for 1 h, and then the remaining PEDV was removed. The PEDV-infected cells were treated with the intracellular extracts of YPK30 or IFN-α2b for 48 h, and then the metabolic activities of Vero cells were determined. As shown in Figure 5, PEDV-infected Vero cells treated with the intracellular extracts of YPK30 for 48 h showed higher cell metabolic activity than the untreated cells or those treated with IFN-α2b (*p* < 0.05), indicating that the intracellular extracts of the YPK30 strain possessed an *in vitro* therapeutic effect against PEDV.

### *In vitro* Direct-Inhibitory Effects of *Ln. mesenteroides* YPK30 Intracellular Extract Against PEDV

**Figure 6:**
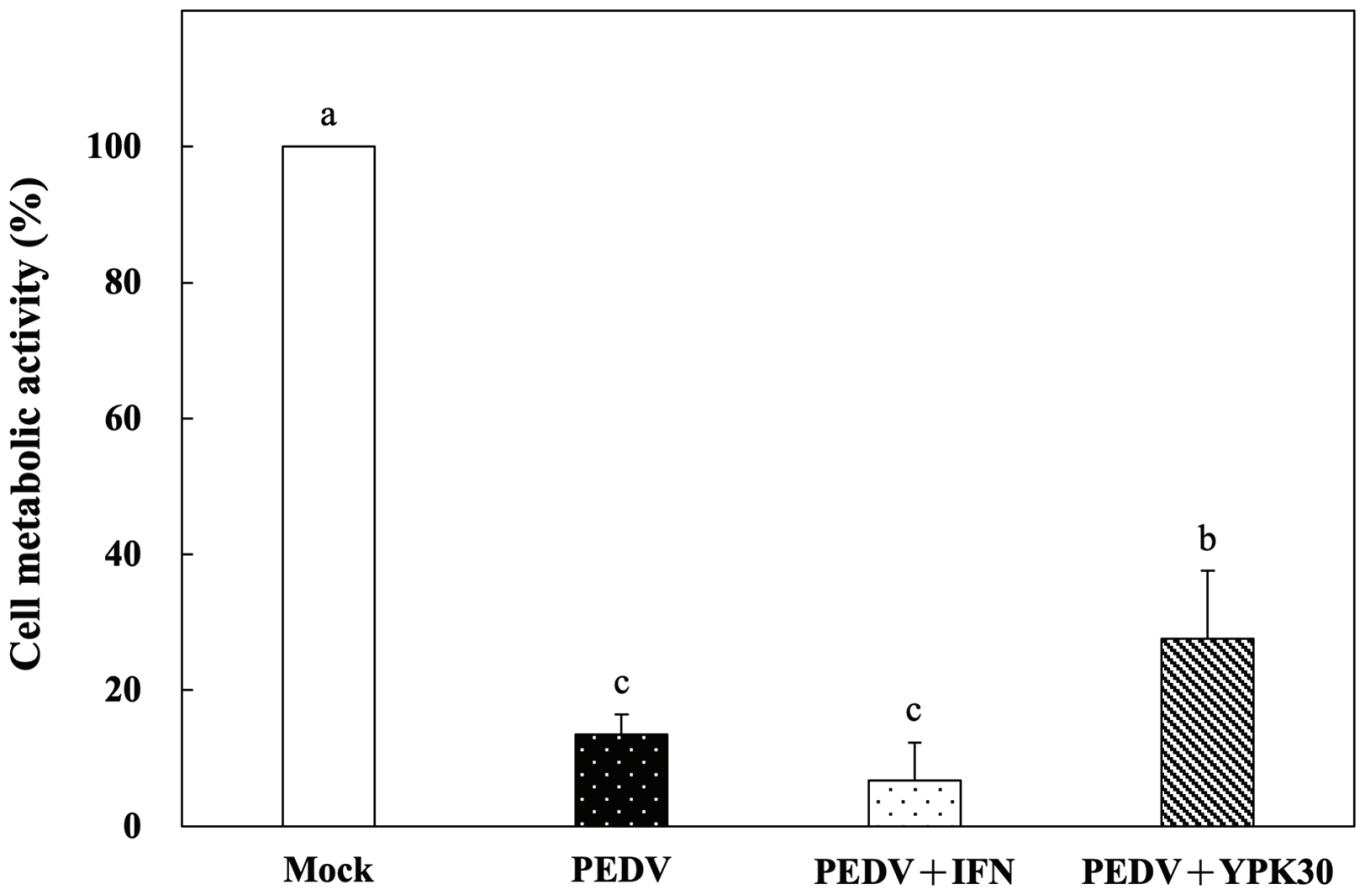
*In vitro* direct-inhibitory effects of the intracellular extracts of *Ln. mesenteroides* YPK30 against PEDV infection in Vero cells. Bar chart showing percentage of cell metabolic activity of four sample conditions: (1) Mock: cells incubated with PBS, (2) PEDV: cells infected with PEDV, (3) PEDV+IFN: cells infected with PEDV and simultaneously treated with IFN-α2b, and (4) PEDV+YPK30: cells infected with PEDV and simultaneously treated with the intracellular extracts of *Ln. mesenteroides* YPK30. All data are expressed as mean ± SD (*n* = 3). Bars marked with the same letter are not significantly different (*p* > 0.05).

Vero cells were co-incubated with PEDV and the intracellular extracts of YPK30 or IFN-α2b for 1 h. After removal of the PEDV and intracellular extracts of YPK30, the Vero cells were incubated for 48 h, and then the metabolic activities of the Vero cells were determined. The metabolic activities of the Vero cells treated with the intracellular extracts of YPK30 and PEDV simultaneously were significantly higher than those treated with PEDV alone or those treated with PEDV and IFN-α2b (Figure 6; *p* < 0.05). These results suggested that the intracellular extracts of YPK30 possessed an *in vitro* direct-inhibitory effect against PEDV in Vero cells.

### Effect of *Ln. mesenteroides* YPK30 Intracellular Extract on Expression Levels of Type 1 IFN-Dependent Genes

**Figure 7:**
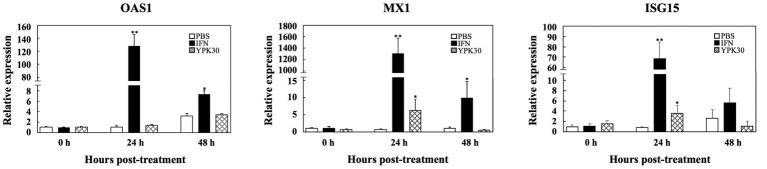
Effect of treatment of intracellular extracts of *Ln. mesenteroides* YPK30 on the expression levels of Type 1 IFN-dependent genes OAS1, MX1, or ISG15 in Vero cells. Bar charts for each gene showing percentage of cell metabolic activity of three sample conditions: (1) PBS: cells treated with PBS, (2) IFN: cells treated with IFN-α2b, and (3) YPK30: cells treated with the intracellular extracts of *Ln. mesenteroides* YPK30. All data are expressed as mean ± SD (*n* = 3). Bars marked with a star or double stars mean that they are significantly different from the control (cells treated with PBS) at the 5 or 1% confidence level, respectively.

In order to elucidate the antiviral mechanisms of the YPK30 strain, the expression levels of Type 1 IFN-dependent genes, including *OAS1*, *MX1*, and *ISG15*, were quantified in Vero cells after 0, 24, or 48 h of treatment with the intracellular extracts of YPK30. As shown in Figure 7, IFN-α2b, which served as the positive control, significantly increased the expression levels of *OAS1*, *MX1*, and *ISG15* genes in Vero cells at 24 and 48 h. The intracellular extracts of YPK30 also significantly increased the expression levels of *MX1* and *ISG15* genes but did not affect the expression levels of the *OAS1* gene in Vero cells at 24 h. However, the expression levels of *OAS1*, *MX1*, and *ISG15* genes in Vero cells did not differ between the untreated and YPK30-treated groups at 48 h.

### Identification of *Ln. mesenteroides* YPK30

According to the 16S rRNA and *rpo*A gene sequence analysis, YPK30 exhibited 99.93 and 99.57%, respectively, identity with *Ln. mesenteroides* (Table 2), and its 16S rRNA and *rpo*A gene sequences were deposited in the NCBI GenBank database under accession number MT293805 and MT333858, respectively. Macroscopic observation showed that YPK30 exhibited a smooth and grayish white colony morphology and did not possess hemolytic capacity on blood agar plate. The cells of YPK30 appeared purple after Gram staining, indicating the strain YPK30 was Gram positive. Microscopic observation showed the cells of YPK30 were observed as spherical or lenticular forms. Analysis on the basis of phenotypic (including Gram-stain-positive, catalase-negative, nonmotile, and asporogenous) and physiological characteristics (including growth at 10 or 37°C but not growth at pH 4.8 or in 10% ethanol) indicated that YPK30 was closely related to species *Ln. mesenteroides*. To further confirm the identification of YPK30 with the species *Ln. mesenteroides*, the biochemical characteristics of YPK30 were compared with those of *Ln. mesenteroides* subsp. *cremoris* ATCC 19254, *Ln. mesenteroides* subsp. *dextranicum* ATCC 19255, and *Ln. mesenteroides* subsp. *mesenteroides* ATCC 8293 by using the API 50 CH system. Analysis of carbon source utilization profiles indicated that both YPK30 and *Ln. mesenteroides* subsp. *dextranicum* ATCC 19255 grew on six out of 49 carbohydrates, including *N*-acetyl glucosamine, D-fructose, D-glucose, D-mannose, saccharose, and D-trehalose. Distinct variation was observed between YPK30 and *Ln. mesenteroides* subsp. *cremoris* ATCC 19254 in the metabolism of the sugars D-fructose, D-mannose, and D-trehalose. Additionally, distinct variation was observed between YPK30 and *Ln. mesenteroides* subsp. *cremoris* ATCC 19254 in the metabolism of the carbohydrates amygdaline, L-arabinose, cellobiose, esculine, D-galactose, β-gentiobiose, D-lactose, maltose, α-methyl-D-glucoside, D-raffinose, ribose, D-turanose, and D-xylose. Therefore, the carbon source use characteristics of YPK30 were similar to those of *Ln. mesenteroides* subsp. *dextranicum*. According to the results of microscopic observations, biochemical characteristics, and the 16S rRNA and *rpo*A gene sequence analysis, the features of YPK30 were consistent with those of *Ln. mesenteroides* subsp. *dextranicum*, as described in Bergey’s Manual of Systematic Bacteriology (Vos et al., 2009).

## Discussion

The Vero cell line is one of the most commonly used cell lines for PEDV isolation and propagation (Koonpaew et al., 2019). In this study, we used a Vero cell culture model to evaluate the *in vitro* prophylactic effects of LAB against PEDV. Four LAB species, including *E. durans*, *Lb. kefiri*, *Lc. lactis*, and *Ln. mesenteroides*, were isolated from kefir grains, and the *in vitro* prophylactic effects of the intracellular extracts of these four species against PEDV infection in Vero cells were compared. Among these four LAB species, the intracellular extracts of *Ln. mesenteroides* showed a higher *in vitro* prophylactic effect against PEDV than the other species did (Figure 1). In addition to the *in vitro* prophylactic effect, the intracellular extracts of *Ln. mesenteroides* YPK30 also possessed *in vitro* therapeutic effect and *in vitro* direct-inhibitory effects against PEDV in Vero cells (Figures 4–6). Vero cells have a major deletion in the Type 1 IFN gene cluster, which results in IFN deficiency (Desmyter et al., 1968; Koonpaew et al., 2019). Although Vero cells do not secrete Type 1 IFNs when infected by viruses, they still have the Type 1 IFN receptors and respond normally to Type 1 IFNs. Therefore, Vero cells were widely used to compare virus-mediated IFN antagonism specific to the IFN signaling pathway (Simmons et al., 2010).

In the *in vitro* prophylactic and therapeutic models, the intracellular extracts of *Ln. mesenteroides* YPK30 did not directly interact with PEDV by physical contact. Therefore, the *in vitro* prophylactic and therapeutic effects of the intracellular extracts of *Ln. mesenteroides* YPK30 against PEDV in Vero cells seem not be attributed to the direct interaction of bacterial components or metabolites with virus. Since the IFN pathway is crucial in initiating viral resistance, we suggest that the *in vitro* prophylactic and therapeutic effects of the intracellular extracts of *Ln. mesenteroides* YPK30 against PEDV in Vero cells could be attributed to its effect on the IFN signaling pathway in Vero cells.

Stimulation of innate immune responses by probiotics could be one of the mechanisms responsible for the protection provided by probiotics against viral infection. Other proposed mechanisms include inhibition of virus adsorption and penetration into cells as a result of direct interaction of probiotics and virus, competition between probiotics and virus for epithelial cell receptors, and secretion of metabolites with antiviral activity (Mousavi et al., 2018; Biliavska et al., 2019). Previous studies have shown that specific probiotic bacteria bind to and inactivate rotaviruses and vesicular stomatitis viruses, which lead to blocking of the virus adsorption on the cell (Salminen et al., 2010; Kanauchi et al., 2018). Besides the direct interaction between probiotics and viruses, specific probiotic bacteria could interact with epithelial and mucosal cells and compete with pathogens for attachment to cell receptors, thereby preventing invasion into the cells by a virus (Fernandez-Duarte et al., 2018; Mousavi et al., 2018). In addition, specific probiotic bacteria could synthesize some antiviral metabolites, such as lactic acid, hydrogen peroxide, or bacteriocins (Al Kassaa et al., 2014). In the present study, the intracellular extracts of *Ln. mesenteroides* YPK30 possessed an *in vitro* direct-inhibitory effect against PEDV in Vero cells (Figure 6). Future studies will be aimed at identifying the mechanisms of the *in vitro* direct-inhibitory effect of the intracellular extracts of *Ln. mesenteroides* YPK30 against PEDV in Vero cells.

Numerous mechanisms for the immunomodulatory properties of probiotics have been proposed. Some extracellular polysaccharides produced by specific probiotic bacteria possess immunomodulatory activities, which induce an increase in the expression of IFN-α, IFN-β, and the antiviral factors MX1 and RNase L in porcine intestinal epithelial cells (Kanmani et al., 2018). Beside extracellular polysaccharides, some cellular components, such as DNA and bacterial cell-wall components, including peptidoglycan, S-layer proteins, teichoic acids, capsule, and pellicle, as well as other released peptides could modulate the innate antiviral immune response (Quinteiro-Filho et al., 2015). In the present study, pretreatment of Vero cells with the extracellular supernatants or intracellular extracts of *Ln. mesenteroides* YPK30 for 24 h before infection with PEDV showed higher cell metabolic activities than those of un-pretreated cells, indicating that both of the intracellular extracts and extracellular supernatants of *Ln. mesenteroides* YPK30 possessed *in vitro* prophylactic effects against PEDV in Vero cells. However, pretreatment of PEDV cells with the cell-wall fractions of *Ln. mesenteroides* YPK30 for 24 h before infection with PEDV did not impede PEDV replication. According to these observations, we suggested that the immunomodulatory activity of *Ln. mesenteroides* YPK30 seems not to rely on the structural cell components. Future studies should be aimed at assessing the molecular mechanism(s) responsible for the observed effects.

Type 1 IFNs exert their antiviral activities though the induction of hundreds of ISGs (Lenschow et al., 2005). Classical ISGs responsible for inhibition of viral infection include OAS1, MX1, and ISG15 (Schoggins, 2014). OAS1, which belongs to the OAS enzyme family, is activated by double-stranded RNA binding, catalyzes the formation of 2′–5′ oligoadenylates to activate cellular RNase L, which in turn, degrade cellular and viral RNA (Choi et al., 2015). MX1 is a dynamin-like GTPase that appears to target viral nucleocapsids, resulting in the inhibition of viral RNA polymerase activity, effectively blocking both transcription and replication of the virus (Schoggins, 2014). ISG15 is a small, ubiquitin-like molecule that has numerous antiviral functions, including inhibition of virus release, ISGylation of both viral and host proteins, and immunomodulatory cytokine-like properties in its unconjugated form (Schoggins, 2014). In the present study, we determined the effects of intracellular extracts of *Ln. mesenteroides* YPK30 on the expression levels of *OAS1*, *MX1*, and *ISG15* genes in Vero cells and found that treatment of Vero cells with the intracellular extracts of *Ln. mesenteroides* YPK30 did not affect the expression levels of the *OAS1* gene but significantly increased the expression levels of *MX1* and *ISG15* genes 24 h after treatment (Figure 7), indicating that the anti-PEDV activity of the intracellular extracts of *Ln. mesenteroides* YPK30 could be attributed to its up-regulatory effect on the expression of *MX1* and *ISG15* genes in Vero cells.

According to the results of microscopic observations, biochemical characteristics, and the 16S rRNA and *rpo*A gene sequence analysis, YPK30 was identified as *Ln. mesenteroides* subsp. *dextranicum*. *Ln. mesenteroides* are commonly associated with foods, such as fermented dairy products (e.g., cheese, yogurt, and kefir), fermented vegetables (e.g., sauerkraut and kimchi), and fermented meats (Holland and Liu, 2011). The long history of safe consumption of *Ln. mesenteroides* in traditional fermented foods has led to the conclusion that it is generally regarded as safe (GRAS; Flórez et al., 2016). Several *Ln. mesenteroides* strains are reported to have anti-listerial, antiviral, or immunomodulatory activities (Seo et al., 2012; Shao et al., 2020). Since the production of exopolysaccharides and bacteriocins are important properties of *Ln. mesenteroides*, the probiotic characteristics of *Ln. mesenteroides* could be attributed to their production of exopolysaccharides and bacteriocins. Several studies demonstrated that some bacteriocins produced by *Ln. mesenteroides* have anti-pathogenic activities (Stiles, 1994; Holland and Liu, 2011; Arakawa et al., 2016), and some exopolysaccharides produced by *Ln. mesenteroides* showed antiviral and immunomodulatory activities (Nácher-Vázquez et al., 2015; Mahdi et al., 2019). In our study, we found that the intracellular extracts of *Ln. mesenteroides* YPK30 possessed *in vitro* prophylactic, therapeutic, and direct-inhibitory effects against PEDV in Vero cells, which occur in part through the up-regulation of *MX1* and *ISG15* expression in Vero cells. To the best of our knowledge, based on a thorough review of the relevant literature, this scientific report is the first of anti-PEDV potential for *Ln. mesenteroides*. Future research will be conducted to evaluate the protection efficiency of *Ln. mesenteroides* YPK30 against PEDV infections in piglet infectious challenge models.

## Conclusion

The LAB strain YPK30 isolated from kefir grains was genotypically and phenotypically characterized as belonging to *Ln. mesenteroides* subsp. *dextranicum*. *Ln. mesenteroides* subsp. *dextranicum* YPK30 displayed *in vitro* prophylactic, therapeutic, and direct-inhibitory effects against PEDV in Vero cells *via* up-regulation of *MX1* and *ISG15* expression in Vero cells. These findings suggest that *Ln. mesenteroides* subsp. *dextranicum* YPK30 has a potential to acts as an antiviral agent for protection against PEDV infections.

## Data Availability Statement

The datasets presented in this study can be found in online repositories. The names of the repository/repositories and accession number(s) can be found in the article/supplementary material.

## Author Contributions

J-RL, W-PC-L, and H-WC contributed to conception and design of the study. W-PC-L, AL, and Y-HC carried out the experiments and did the data analysis. W-PC-L and J-RL wrote the first draft of the manuscript. All authors contributed to the manuscript revision, read and approved the submitted version. The corresponding author takes primary responsibility for communication with the journal and editorial office during publication.

## Conflict of Interest

The authors declare that the research was conducted in the absence of any commercial or financial relationships that could be construed as a potential conflict of interest.
